# Treatise on Diseases of Infants and Children

**Published:** 1881-08

**Authors:** 


A TREATISE ON DISEASES PECULIAR TO INFANTS AND CHILDREN. By *W. A. Edmonds, M. D.*, Professor of Pædology in the St. Louis Homœopathic College of Physicians and Surgeons, etc., etc. pp. 300, price $2.50. Bœricke & Tafel: New York and Philadelphia. London: Trübner & Co., Ludgate Hill. Homœopathic Publishing Co., 2 Finsbury Circus. 1881.

The author commences his task with some good advice on the hygiene, etc., of infancy and childhood. Especially excellent is his advice on the mental culture of the child. He believes the "first ten years should be given to the culture of arms, legs, bone and muscle, as the best guarantee for that most desirable state of 'sound mind in a sound body.'"

The description of the many diseases peculiar to infants and children is good, and quite complete for a small work. As homœopathic physicians, it is chiefly the therapeutics of the subject in which we are interested; though nowhere is hygiene more important than in the subjects treated of in this book, and *must* never be overlooked.

*Therapeutics:* At first glance it is evident that our author believes in "strong medicine." His doses are large, and entirely too frequent. While we believe in the greater efficacy of the high potencies—that they will cure where the low fail, yet we do not condemn the use of the low. Those whose confidence or faith in the power of dilutions is too weak to allow them to use the high, should, we think, use the low; but no judicious homœopathic practitioner can find reason or excuse for profuse dosing. It is not the quantity, but the appropriate quality of the medicine, that does the healing. As an instance of his heavy dosing, on page 152 our author recommends for vomiting remaining after a diarrhœa: "in such an emergency kreosote at the 2 x dilution, in drop doses, every ten, twenty or thirty minutes, on a little crushed ice, will be found a most valuable resource." "A mild sinapism over the epigastrium" is also advised for the same symptom. In the treatment of cholera infantum, he says, "the list of remedies is small, but the result prompt, complete and brilliant." That "the list of remedies is small" will be news to all who use Dr. Bell's book, with its clear indications for 140 remedies.

Alternation—a bad practice—is frequently advised. Thus, at page 28, *Bell.* and *Merc.* are recommended in alternation for mammary swelling in the new-born infant; at page 100, in speaking of croup, he says: "*Tartar emetic* alternates well with *Aconite* from the start;" at page 153, *Merc. Dulcis* and *Ipecac.* for cholera infantum with bilious vomiting; at page 265, we read: "for obstinate chronic *furunculus,* I know of no prescription so satisfactory as *Sulphur* and *Belladonna* in alternation."

For the severe *pruritus:* "℞.—Chloral hydrate, . . . . . grs. x

Aqua, . . . . . . . . ℥ j

Glycerin, . . . . . . . ʒ j

Fiat lotio. Sig.: Moisten parts three or four times per day."

For an extensive and violent eczema, he orders: "Arsenicum 2x, internally, one grain, three times per day, and the local application of an ointment, composed of one ounce of Vaselin to ten grains of Oxide of Zinc, to be applied three times daily."

These few quotations are given that the bad advice of our author may be clearly noted. The indications for the remedies, mentioned throughout the book, are scant and indefinite. In conclusion, we would say that the practice advocated in this book differs very decidedly from that taught us by Hahnemann. Such books are an injury to homœopathy.

